# From Chronic Cannabis to Cyclic Chaos: A Glimpse Into Cannabinoid Hyperemesis Syndrome

**DOI:** 10.7759/cureus.64828

**Published:** 2024-07-18

**Authors:** Omar Oraibi

**Affiliations:** 1 Department of Internal Medicine, Jazan University, Jazan, SAU

**Keywords:** periodic vomiting, nausea, hyperemesis, cannabinoid, abdominal pain

## Abstract

Cannabinoid hyperemesis syndrome (CHS) pathophysiology remains largely unknown, and it is often misdiagnosed. This paper identifies the clinical causes of CHS, outlines diagnostic and therapeutic approaches, and emphasizes early detection, comprehensive treatment, and timely intervention for improved patient outcomes. This case describes a 38-year-old male with a known history of cannabis use who experienced repeated episodes of intense vomiting, nausea, and abdominal pain consistent with symptoms of CHS. He was initially misdiagnosed with other gastrointestinal conditions despite the knowledge of marijuana ingestion. The diagnosis of CHS was initially missed; however, after further examination and consideration of his marijuana use, CHS was correctly identified. The patient's symptoms improved after the cessation of marijuana use. This case illustrates the diagnostic difficulties of cannabinoid hyperemesis syndrome (CHS) in cannabis users with significant gastrointestinal symptoms. The early detection and cessation of marijuana use are crucial for symptom management and resolution, emphasizing clinical awareness and personalized treatment.

## Introduction

The recreational use of marijuana, also known as cannabis, has become increasingly prevalent in recent years, in part due to its legalization in numerous countries. This has led to a significant shift in the social and legal perception of cannabis, with more countries reevaluating their marijuana laws [1].

In addition to marijuana's recreational use, it has also been recognized for its medical applications, including its efficacy as an antiemetic and appetite stimulant. Given its potential therapeutic benefits, it has been the focus of significant research in recent years, with scholars examining its effects on a variety of medical conditions [1,2]. Though its use remains controversial, the growing body of evidence suggests that it may have a valuable role to play in modern medicine [3,4]. Furthermore, synthetic cannabinoids are often utilized in palliative care to manage symptoms of pain, nausea, and vomiting. These compounds, which are artificially produced to mimic the effects of natural cannabinoids found in the cannabis plant, are effective in reducing symptoms in patients with advanced illnesses.

While cannabis has demonstrated significant potential in treating various conditions, its extended and continuous use has also been linked to a problematic condition called cannabinoid hyperemesis syndrome (CHS), which is characterized by recurrent episodes of severe nausea, vomiting, and abdominal pain. First described by Allen et al. in 2004, CHS is paradoxical and poorly understood [5].

The patient may experience distress due to the condition, leading to frequent hospital visits and affecting both the patient's quality of life and healthcare costs [6]. One of the notable complications associated with CHS is electrolyte imbalance, particularly hypokalemia and hyponatremia, which can result from persistent vomiting and inadequate fluid intake [7,8]. These electrolyte disturbances can lead to significant morbidity, including cardiac arrhythmias, muscle weakness, and renal dysfunction [9].

With cannabis being easily accessible and having a high potential for abuse, CHS is a widespread health concern. It has been challenging to find effective antiemetic treatments for CHS due to the similarity of symptoms with other conditions such as cancer, chemotherapy, cyclic vomiting syndrome, viral gastroenteritis, and bulimia nervosa [2]. This report seeks to raise awareness about the role of cannabis in the development and management of CHS.

## Case presentation

A 38-year-old male with a known history of cannabis use presented to the clinic with several episodes of severe nausea, vomiting, and severe abdominal pain, which had affected his daily activities for the previous 3-4 years. The patient reported that he had frequent episodes of vomiting, up to six to eight times a day, associated with severe nausea and colicky and nonradiating epigastric abdominal pain. During this visit, the patient reported that he could not retain any food or fluid and had lost about 8 kg the previous year. Each episode lasted about two days. The patient reported no known significant medical history. He denied taking any prescribed medications and had no known allergies. There was no history of fever, chills, hematemesis, hematochezia, constipation, diarrhea, heartburn, or dysphagia. He denied recent travel or exposure to individuals with known illnesses.

He reported that he had experienced similar attacks over the previous few years associated with life stressors, requiring multiple emergency department and outpatient clinic visits. There is no family history of a similar illness. He reported a long history of cannabis use but no use of alcohol or other illicit drugs. He mentioned that he used to consume cannabinoid sticks daily, which was not permitted, typically about five to six sticks per day.

During the patient's visit to the clinic, the medical examination noted that the patient's vital signs were within normal parameters. It revealed no significant abnormalities except mild tenderness in the epigastric region upon palpation. There was no sign of bowel obstruction. The patient received a comprehensive workup, including laboratory tests, imaging testing, esophagogastroduodenoscopy, and coloscopy. A complete blood count with differential, lipase, amylase, glycated hemoglobin, and urine analysis were done (Table 1). A comprehensive metabolic panel was within normal limits except for electrolyte imbalances, mild hyponatremia, and hypochloremia resulting from prolonged vomiting (Table 2). No other significant medical complications were observed.

**Table 1:** Laboratory parameters of the patient

Parameter (unit)	Laboratory values	Reference range
White cell count, total (×10^3^/µL)	7.3	4.5-11
Hemoglobin, serum (g/dL)	13.7	12-16
Lipase, serum (U/L)	12	0-160
Amylase, serum (U/L)	24	30-110
Glycated hemoglobin (HbA1c) (%)	5.1	<5.7

**Table 2:** Metabolic panel of the patient

Parameter (unit)	Laboratory values	Reference range
Creatinine, serum (mg/dL)	0.93	0.6-1.2
Urea nitrogen, serum (mg/dL)	8	5-20
Sodium, serum (mEq/L)	134	135-145
Potassium, serum (mmol/L)	4.9	3.6-5.2
Chloride, serum (mEq/L)	93	96-106
Calcium (mg/dL)	9.3	8.5-10.5
Aspartate aminotransferase (U/L)	44	<40
Alanine aminotransferase (U/L)	34	<40

**Figure 1:**
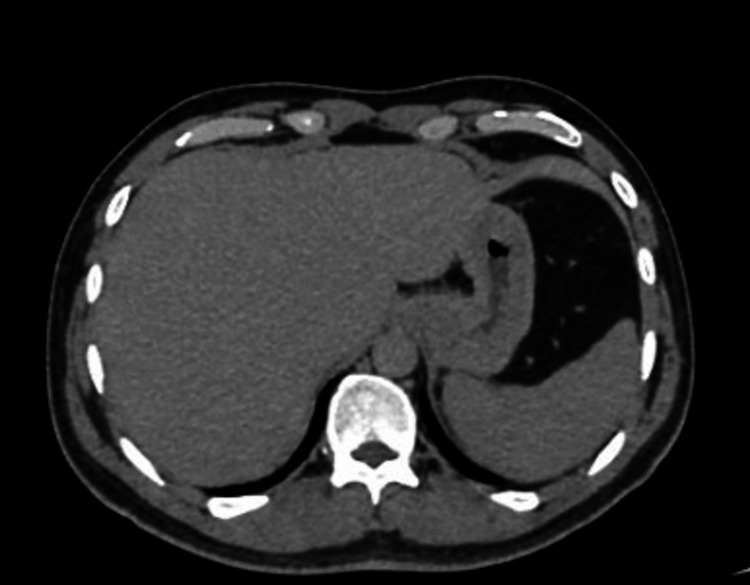
Normal abdominal CT scan CT: computed tomography

Additionally, the urea breath test was negative (Table 3). Additional tests, an abdominal X-ray, computed tomography (CT) of the abdomen, gastric emptying study, gastric biopsies, esophagogastroduodenoscopy, and colonoscopy, were all normal (Figure 1). None of the tests or imaging studies were able to identify a pathology that could explain his recurrent illness.

**Table 3:** Urea breath test result

Test description	Result	Unit	Reference range
Urea breath test (UBT)	1.4	%	Negative, <3.6; positive, >3.6

The patient was diagnosed with cyclic vomiting syndrome and treated for dehydration, nausea, vomiting, and abdominal pain. Intravenous (IV) ondansetron was administered for its antiemetic effect, and a proton pump inhibitor was administered for gastrointestinal prophylaxis and intravenous fluid resuscitation. The patient was counseled to discontinue cannabis use and was given support through educational resources. After being discharged, the patient refrained from using cannabis, and the CHS symptoms disappeared, with no recurrence or new episodes reported after that.

## Discussion

CHS causes recurring episodes of nausea, vomiting, and stomach pain [10]. This condition occurs every few weeks to months and usually goes away after cannabis consumption stops. CHS, which is increasingly recognized yet underreported due to cannabis usage, is difficult to diagnose. When treating cannabis-using patients with recurrent vomiting and stomach pain, professionals should investigate CHS. Early diagnosis and treatment reduce morbidity and improve patient outcomes [10,11].

The underlying mechanism of CHS remains inadequately understood. Particularly puzzling is why a drug typically prescribed for nausea relief sometimes causes severe vomiting. Delta-9-tetrahydrocannabinol (THC) is widely recognized as the most prominent psychoactive constituent of cannabis. The body normally absorbs THC through inhalation via the lungs or ingestion through the gastrointestinal tract, after which it enters the bloodstream [12]. THC attaches to cannabinoid receptors (CB), specifically CB1 and CB2, found in the central nervous system (CNS), gastrointestinal tract, and peripheral tissues. These are evenly distributed throughout the body's tissues [13].

The CNS predominantly comprises the CB1 receptor, which is thought to substantially influence several activities, including memory, mood, brain reward systems, drug addiction, energy balance, and metabolic processes such as lipolysis and glucose metabolism [13].

The THC activation of gastrointestinal CB1 receptors may affect motility, appetite, nausea, and vomiting [7]. Cannabis is also thought to reduce nausea and increase appetite due to CB1's antiemetic properties. This is particularly relevant for patients undergoing chemotherapy or those with chronic illnesses such as HIV/AIDS [9].

Sontineni et al. established a set of diagnostic criteria summarizing the fundamental characteristics of long-term cannabis consumption, which include repetitive and intense episodes of nausea and vomiting that can be alleviated by discontinuing cannabis use. The supportive characteristics of the criteria include mandatory hot baths for symptomatic relief and colicky abdominal pain. These criteria provide a comprehensive framework for the diagnosis of cannabis-induced cyclical vomiting syndrome and can aid healthcare professionals in providing effective treatment to affected individuals [14].

Individuals presenting with CHS typically exhibit prolonged and regular cannabis use, defined as consumption for more than one year and at least once per week, respectively [8,15]. Those with CHS also share common population demographics, such as being male and under 50 years of age [15].

CHS diagnostic testing and a full medical and personal history are performed to rule out alternative explanations of symptoms and evaluate for physical abnormalities. A basic electrolyte examination, blood counts, and imaging studies are done to construct a treatment plan and diagnosis. Abdominal ultrasound, abdominal CT, gastric emptying investigations, and upper and lower gastrointestinal series may be needed to rule out or disprove other diseases. In cases where the abdominal (gastrointestinal) workup is unrevealing, gastrointestinal disorders (e.g., peptic ulcer disease, gastritis, and pancreatitis), CNS pathologies, and rare metabolic disturbances (thyrotoxicosis, porphyria, Addison's disease, etc.) should also be considered in the differential diagnosis [16,17]. Cannabinoids can be detected in urine drug screens to diagnose CHS [15].

The patient with CHS is presently receiving medical care for dehydration, nausea, anxiety, and additional health concerns. The treatment regimen includes intravenous (IV) fluids, anxiolytics, and antipsychotics [7]. At present, the sole recognized course of action for CHS is the cessation of cannabis use [8]. However, the application of topical capsaicin cream of 0.075% to the abdomen, along with the administration of haloperidol 5 mg intravenously or intramuscularly, has proven to be the most effective therapeutic approach for relieving the patient's nausea and vomiting [15,18]. Hot water showers are commonly utilized as standard therapy for CHS due to their significant symptomatic relief [15]. However, caution must be exercised to avoid hot water burns because, despite its benefits, the therapeutic effects of hot water are short-lived [19].

Cannabis cessation can begin quickly in CHS without withdrawal symptoms. Thus, addiction counseling and cannabis paradox education should be part of the treatment [15]. The duration of cannabinoids' half-life differs significantly, particularly in individuals who consume them daily over the long term, resulting in an extended terminal elimination period [20]. Therefore, it is advisable to recommend extended periods of abstinence.

## Conclusions

Given the expected growth in CHS cases, more studies are needed to understand its etiology and develop effective treatments. Healthcare providers should discourage patients from cannabis use and disseminate public health messages regarding cannabis risks. This will help us better serve CHS patients and mitigate its impact on society.
